# Determinants of patient satisfaction following reconstructive shoulder surgery

**DOI:** 10.1186/s12891-017-1812-x

**Published:** 2017-11-15

**Authors:** Sascha J. Baettig, Karl Wieser, Christian Gerber

**Affiliations:** Orthopaedic Department, Balgrist University Hospital, University of Zurich, Forchstrasse 340, 8008 Zurich, Switzerland

**Keywords:** Patient satisfaction, Determinants, Shoulder surgery, Rotator cuff repair, Shoulder arthroplasty, Reconstructive shoulder surgery, Satisfaction, Factors

## Abstract

**Background:**

Obtaining patient satisfaction is a key goal of surgical treatment. It was the purpose of this study to identify pre-, peri- and postoperative factors determining patient satisfaction after shoulder surgery, quantify their relative importance and thereby allow the surgeon to focus on parameters, which will influence patient satisfaction.

**Methods:**

We retrospectively reviewed 505 patients, who underwent either rotator cuff repair (*n* = 216) or total shoulder arthroplasty (*n* = 289). We examined 21 patient-specific and socio-demographic parameters as well as 31 values of the Constant-Score with regard to their impact on patient satisfaction.

**Results:**

In the univariable analysis higher patient satisfaction was correlated with higher age, private health insurance, light physical work, retirement, primary surgery, non-smoking, absence of chronic alcohol abuse, absence of peri- or postoperative complications, operation performed by the medical director as well as various Constant Score sub-values (*p* < 0.05). In the multivariable analysis absence of peri- or postoperative complications (*p* = 0.008), little postoperative pain (*p* = 0.0001), a large range of postoperative active abduction (*p* = 0.05) and a high postoperative subjective shoulder value (*p* = 0.0001) were identified as independent prognostic factors for high satisfaction.

**Conclusion:**

After reconstructive shoulder surgery particular attention should be paid to prevention of complications, excellent perioperative pain control and restoration of abduction during rehabilitation. This study is first step towards a preoperative prediction model of a subjectively successful surgery as well as a tool to exclude irrelevant parameters in clinical routine.

## Background

Subjective outcome parameters such as self-assessment of function, quality of life or patient satisfaction have become fundamental tools for outcome assessment of orthopaedic interventions [1]. Patient satisfaction is a reliable indicator of health care quality, enabling the comparison between different health care providers [2]. Patient reported outcomes may guide patients to choose their health care provider and could substantially influence competition in health care markets [3] [4]. Objective treatment success is essential, but not the only condition, which generates patient satisfaction [5, 6]. Many factors such as age [7–10], gender [7], marital status [11], occupation [8, 9, 11, 12], workers’ compensation status [7–9, 13–17], presence of revision surgery after a previously failed operation [7], preoperative expectations [11], postoperative pain [8, 11, 12, 18, 19] and postoperative range of motion (i.e. internal rotation [8, 12], anteversion/elevation [10, 12, 18, 19] had already been identified to influence patient satisfaction after shoulder surgery. However, the majority of the above mentioned studies had substantial limitations in scope and or validity. But there exist a few other studies with excellent quality, which examine determinants of patient satisfaction after surgery in other articulations for example the knee [20, 21].

Patient satisfaction plays a pivotal and not thoroughly studied role in assessing surgical outcome. The identification of positive and negative predicting factors could lead to preoperative prediction models for determining the probability of an (un-)desired surgical outcome. It appears particularly important also to know parameters, which do not affect patients’ postoperative satisfaction because it may help surgeon and therapist to avoid wasting energy in efforts not leading to improvement of patient satisfaction.

The purpose of this study was to systematically analyse as many allegedly relevant determinants in one evaluation and study their true influence on patient satisfaction following operative treatment of rotator cuff tears or osteoarthritis in a multivariable regression model.

## Methods

### Setting and patient selection

We retrospectively reviewed the shoulder database to identify patients, who had been treated in our institution, either with open or arthroscopic rotator cuff repair or implantation of a total or reverse shoulder arthroplasty between January 1999 and December 2011. In this time period we performed approximately 2500 surgeries, after randomization we selected 600 patients for the evaluation in this study. Patients with prospectively recorded, complete pre- and postoperative Constant Scores (CS), subjective shoulder value (SSV) and a documented postoperative patient satisfaction were included in this study. Out of the 600 selected patients, 505 patients met the mentioned criteria. For a patient with multiple monitoring consultations, we selected the data closest to 24 months after the index surgery for final analysis. Postoperative scores or patients’ satisfaction obtained earlier than 12 or later than 60 months after index surgery were excluded. This study was approved by the Swiss Ethics Committees on research involving humans (KEK-ZH-2014-0377).

Independent factors potentially influencing patient satisfaction extracted from patients’ charts comprised: (1) Age, (2) BMI, (3) gender, (4) marital status, (5) health insurance status, (6) occupation, (7 and 8) affected/dominant side, (9) opposite shoulder affected, (10) nature of injury (labour vs. leisure trauma vs. non-traumatic orthopaedic disease), (11) chronic nicotine abuse (> 10 pack-years), (12) chronic alcohol abuse (male: > 30 g; female: > 20 g/day [22]), (13) clinical position of the responsible surgeon, (14) length of follow-up, (15) number of non shoulder-specific previous operations, (16) Diabetes mellitus, (17) revision or primary surgery, (18) psychopharmacological drug use, (19) immunosuppressive medication, (20) peri- and/or postoperative complications (surgical site infection, iatrogenic neurological lesion, hematoma evacuation, extended intensive care unit stay), (21) chronic comorbidities (symptomatic cardio-vascular disease, COPD, asthma, autoimmune disease, neurologic (Parkinson disease, multiple sclerosis, dementia), infectious disease (HIV, hepatitis A/B), endocrinological diseases, chronic cervical or lumbosacral pain syndrome, gastrointestinal diseases, gout and chronic renal insufficiency). The decisive date for gathering the information for parameters (4, 5, 6, 11, 12, 18, 19, 21) was the time of surgery. In addition, all values of the pre- and postoperative Constant Scores, the amount of improvement (delta) of those values as well as the pre- and postoperative SSV were analysed regarding their implication on patient satisfaction.

### Outcome measure

We identified 505 patients, who underwent either rotator cuff repair (*n* = 216) or shoulder arthroplasty (total or reverse) (*n* = 289) and met the above mentioned inclusion criteria for this investigation. The study comprised 271 men and 234 women. The age of the 505 patients ranged from 17 to 90 years old (mean: 61.4 years, SD: 12.3 years). The surgery had involved 71% dominant shoulders. The mean time period between surgery and the measurement of the patients’ satisfaction was 23.6 months (range: 12–60 months) (Table 1). Our primary target variable was patient satisfaction, which was documented at the postoperative consultation closest to 24 months after index surgery. The patients graded their satisfaction as: not satisfied, rather dissatisfied, rather satisfied and very satisfied (Table 2).

**Table 1:** Patients‘Characteristics

Variable	Data
Number of patients analysed in total	505
Mean age of the patient collective	61.4 years (range 17–90 years)
Mean time period between surgery and satisfaction measurement	23.6 months
Gender	Male = 271, Female = 234
Family Status	Married = 328, Single = 169
Number of patients retired	194 (38.4%)
Number of patients with a traumatic origin of injury	340 (67.3%)
Mean BMI of the patient collective	26.9 (range 15.7–48.0)
Number of patients smoking	89 (17.6%)
Affected side	Right = 339, Left = 166
Number of patients with the dominant shoulder involved	358 (70.9%)
Mean preoperative Constant Score	40.4 (range 2.0–100.0) points
Mean postoperative Constant Score	59.6 (range 3.0–100.0) points

**Table 2:** Patients’ satisfaction

Patients’ satisfaction	Rotator cuff reconstruction	Arthroplasty
Highly satisfied patients	60 (27,8%)	122 (42.2%)
Rather satisfied patients	50 (23.1%)	80 (27.7%)
Rather dissatisfied patients	52 (24.1%)	57 (19.7%)
Dissatisfied patients	54 (25%)	30 (10.4%)
	216 (100%)	289 (100%)

### Statistical methods

Statistical analysis was performed under the supervision of an experienced biomedical statistician using IBM SPSS Statistics, Version 21.0, Armonk, NY. The level of significance was set at (*p* < 0.05). Due to the numerous statistical comparisons we calculated an additional Bonferroni-corrected level of significance (*p* = 0.00095). To increase the significance and validity of the evaluation particularly with regard to the multivariable regression model we divided the patients in a satisfied (including rather satisfied and very satisfied, *n* = 312) and dissatisfied (including rather dissatisfied and not satisfied, *n* = 193) group. Pearson Chi squared test was performed for categorical determinants in the univariable analysis. The effect of the numeric values was analysed with the non-parametric test of Mann and Whitney. Univariable significant variables were integrated in a logistic regression dependent on likelihood-quotients (calculated backwards), to identify independent prognostic factors of patients’ satisfaction. For the delta of score sub-values a multivariable analysis was not accurate due to its dependency on pre- and postoperative values.

## Results

### Univariable analysis

The univariable analysis revealed that, higher age (*p* = 0.0001), private health insurance (p = 0.0001), primary surgery (p = 0.0001), non-smoking (*p* = 0.001), absence of intra- or postoperative complications (p = 0.001) and the absence of chronic alcoholism (*p* = 0.039) were associated with high patient satisfaction. Retired patients or patients with light physical work were significantly more satisfied than manual workers and especially significantly more than patients, who were job seeking or had on-going workers compensation claims (*p* = 0.0001). Furthermore, patients treated by the medical director showed a higher satisfaction than patients treated by a senior surgeon or senior consultant (*p* = 0.045). Pursuant to our results there was no significant correlation between patients satisfaction and BMI (*p* = 0.886), gender (*p* = 0.238), marital status (*p* = 0.442), affected side (*p* = 0.502), dominance (*p* = 0.521), opposite shoulder affected (*p* = 0.363), nature of the injury (*p* = 0.139), more than 2 previous non shoulder-specific operations (*p* = 0.548), chronic comorbidity (*p* = 0.382), diabetes mellitus (*p* = 0.171), regular consumption of psychotropic drugs (*p* = 0.741) and immunosuppressive medication (*p* = 0.177). Compared to the “Bonferroni-corrected” level of significance (*p* = 0.00095) following determinants forfeit their significance: non-smoking, the absence of chronic alcoholism, the absence of intra- and postoperative complications and the status of the responsible surgeon (Tables 3 and 4).

**Table 3:** Results

Univariable analysis				Multivariable
Determinant	Number of pat.	Satisfied patt. *	*p*-value	*p*-value
Type of operation			0.0001**	0.0001
Rotator cuff	216	51%		
Totalarthroplasty	289	73%		
Gender			0.238	n.s.
Male	271	59%		
Female	234	65%		
Marital status			0.442	n.s.
Single	169	64%		
Married	328	60%		
Health insurance			0.0001^**^	n.s.
Statutory	226	53%		
Private	266	69%		
Occupation			0.0001^**^	n.s.
Light physical work	195	64%		
Heavy physical work	90	47%		
Retired	194	69%		
Job-seeking/IV-Ins.	22	36%		
Affected side			0.502	n.s.
Right	339	61%		
Left	166	64%		
Dominance			0.521	n.s.
Dominant	358	61%		
Adominant	147	64%		
Opposite shoulder affected			0.363	n.s.
No	428	63%		
Yes	77	57%		
Nature of the injury			0.139	n.s.
Trauma during leisure time	294	59%		
Trauma during labour time	46	61%		
Non-traumatic orthopaedic disease	165	68%		
Revision surgery			0.0001^**^	0.062^***^
Yes	225	49%		
No	280	72%		
Non shoulder-specific previous operations			0.548	n.s.
Less than 2	306	61%		
More than 2	197	63%		
Smoking			0.001	n.s.
No	416	65%		
Yes	89	46%		
Alcohol abuse			0.039	n.s.
No	468	63%		
Yes	37	46%		
Responsible surgeon			0.045	n.s.
Senior surgeon	70	61%		
Senior consultant	137	53%		
Medical director	298	66%		
Chronic Comorbidity			0.382	n.s.
No	371	61%		
Yes	134	65%		
Diabetes mellitus			0.171	n.s.
No	475	63%		
Yes	30	50%		
Psychotropic drugs			0.741	n.s.
No	417	62%		
Yes	88	60%		
Immunosuppressive medication			0.177	n.s.
No	478	61%		
Yes	27	74%		
Complications (intra-,postOP)			0.001	0.008
No	459	64%		
Yes	46	39%		

^*^Contains only very satisfied and satisfied patients

^**^Significant compared to “Bonferroni-corrected” p-value (*p* = 0.00095)

^***^Without significance

**Table 4:** Result Scores

Determinant	Average value	Higher satisfaction with:	*p*-value	Multivariable Analysis
Age	61.4	higher	0.0001^**^	n.s.
Length of follow-up	23.6		0.226	
BMI	26.9		0.886	n.s.
Preoperative Constant Score				
Pain (0–15 pts.)	6.2	higher ^*^	0.45	n.s.
Activities of daily living (0–10 pts.)	4	higher	0.0001^**^	n.s.
Reach of the hand (0–10 pts.)	6.2		0.857	n.s.
Anteversion (0–10 pts.)	5.5		0.901	n.s.
Abduction (0–10 pts.)	4.9		0.857	n.s.
External rotation (in degrees)	30.4	higher	0.008	n.s.
Internal rotation (in degrees)	29.7		0.423	n.s.
Force (0–25 pts.)	3.7		0.628	n.s.
Total CS (0–100 pts.)	40.4		0.331	n.s.
Total CS, age-adjusted (%)	50		0.05	n.s.
Preoperative subjective shoulder value (%)	36.3	higher	0.0001^**^	n.s.
Postoperative Scores				
Pain (0–15 pts.)	10.5	higher ^*^	0.0001^**^	0.0001
Activities of daily living (0–10 pts.)	6.9	higher	0.0001^**^	n.s.
Reach of the hand (0–10 pts.)	8.2	higher	0.0001^**^	n.s.
Anteversion (0–10 pts.)	7.1	higher	0.0001^**^	n.s.
Abduction (0–10 pts.)	6.8	higher	0.0001^**^	0.05
External rotation (in degrees)	35		0.118	n.s.
Internal rotation (in degrees)	22.6		0.982	n.s.
Force (0–25 pts.)	6.8	higher	0.0001^**^	n.s.
Total CS (0–100 pts.)	59.6	higher	0.0001^**^	n.s.
Total CS, age-adjusted (%)	74.2	higher	0.0001^**^	n.s.
Postoperative subjective shoulder value (%)	63.3	higher	0.0001^**^	0.0001
Changes Constant Score				
Pain	4.3		0.0001^**^	***
Activities of daily living	2.9		0.0001^**^	***
Reach of the hand	2		0.0001^**^	***
Anteversion	1.1		0.0001^**^	***
Abduction	1.9		0.0001^**^	***
External rotation	4.6		0.0001^**^	***
Internal rotation	−7.1		0.049	***
Force	3.1		0.0001^**^	***
Total Constant Score (Pts.)	19.2		0.0001^**^	***

^*^A high pain score means low effective pain in the measurement of the Constant Score

^**^Significant compared to “Bonferroni-corrected” *p*-value (*p* = 0.00095)

^***^Multivariable analysis is not possible

The analysis of the *pre*operative (Constant) score identified the following factors to be associated with higher patient satisfaction: higher activities of daily living (*p* = 0.0001), higher preoperative subjective shoulder value (p = 0.0001) and a higher range of external rotation (*p* = 0.008). All constituent parts of the *post*operative Constant Score (including postoperative subjective shoulder value) had an influence on higher patient satisfaction except the range of the internal (*p* = 0.118) and external rotation (*p* = 0.982). Lastly, a higher improvement (delta) of the Constant Score had a positive influence on patient satisfaction. Compared to the “Bonferroni-corrected” level of significance (*p* = 0.00095) the preoperative age-adjusted Constant Score, the preoperative range of external rotation and the improvement of internal rotation loose their significance. (Table 4).

### Multivariable analysis

Multivariable analysis retained as independent determinants: absence of intra- or postoperative complications (*p* = 0.008), low postoperative pain (*p* = 0.0001), high postoperative subjective shoulder value (p = 0.0001) and a large range of postoperative abduction (*p* = 0.05). Primary surgery (*p* = 0.062) and a high range of preoperative external rotation (*p* = 0.064) showed a trend to higher satisfaction but missed the level of significance (Tables 3 and 4).

## Discussion

Subjective outcome research has become much more relevant over the last decades [11]. A key capacity of patient satisfaction is the opportunity to critically assess medical outcome or treatment methods. It offers new tools to compare procedures and health care providers or enables the validation of health care quality of an existing environment [8]. The purpose of this study was to investigate how patient satisfaction after a rotator cuff repair or an implantation of a shoulder arthroplasty is composed and to establish a list with all determinants of ultimate patient satisfaction an orthopaedic surgeon should consider. We confirmed our hypothesis and identified various determinants and score values, which are associated with patient satisfaction.

Despite a rather large study population of 505 patients we are aware of potential limitations of this study. First this is a retrospective study of data, which were prospectively collected in a standardized fashion. Patients who wish or consent to undergo surgery may be more positive than those who have elected not to be operated on and who are not included in this study. Further, we selected patients with complete data sets: this may contain a bias as patients not reporting back may have other perceptions of satisfaction. However, overall satisfaction of our population is compared to other studies rather reduced [18] and as well to other orthopaedic interventions [23]. This fact is probably related with the selection criteria because dissatisfied Patient had more frequent consultations and more complete data. Further, we are aware of other potential influencing factors like orthopaedic disease, type of surgery, which we excluded on purpose from the analysis as we really focused on other independent factors influencing the patients satisfaction.

Despite these limitations we were able to identify factors, which showed neither in the multi- nor univariable analysis any influence on patients satisfaction:

*Gender* does not play a relevant role in the determination of patient satisfaction. This fact is agreed upon in the orthopaedic literature for rotator cuff repairs [9, 11, 12, 24], implantations of hemi- and total arthroplasties [8] or shoulder stabilisations [19, 25]. In addition, our findings are in accordance with the results of the study of Tashjian et al. [11], which shows that *marital status* is not a relevant determinant of patient satisfaction after rotator cuff repair. Furthermore, we found in our evaluation that the *affected side*, the *dominance*, the case of *both shoulders affected* or the *nature of the injury* does not play a decisive role for the patients’ postoperative satisfaction. This result corresponds with the result of Kim et al. [9] concerning the determinant dominance of the affected shoulder.

It has been postulated in literature that psychosocial factors, especially preoperative psychological distress, such as depression, is associated with poor clinical outcome [26–28]. We have tried to incorporate various psychiatric diagnoses in a single variable and investigated the effect of psychotropic drugs on patient postoperative satisfaction. A correlation between those variables could, however, not be confirmed in our evaluation. In addition, we scrutinized the potential effect of different *chronic comorbidities*. In accordance with the results of Tashjian et al. [11] and Jacobs et al. [18] we were unable to find a correlation. Pursuant to our knowledge, this is the first study, which has analysed specific determinants like the presence of *immunosuppressive medication* or *non-orthopaedic previous surgeries*. However, none of these determinants showed an influence on the resulting satisfaction.

Despite all other sub-values of the postoperative Constant score, neither *postoperative internal- nor external rotation* showed a correlation with higher patient satisfaction. Although previous reports did show a positive correlation between internal rotation and patients satisfaction [8, 12], postoperative external rotation was also found to be of little importance regarding patients satisfaction [12, 18, 19]. A possible explanation for the unexpectedly missing correlation between patients’ satisfaction and internal and external rotation might be the fact, that it is not the maximum amount of rotation but the absence of a necessary minimal achievement, which indisputably will influence patient satisfaction.

Furthermore this investigation revealed factors, which seem to have some influence on patients satisfaction (significant influence in univariable analysis) but might be confounded and influenced by other factors (no significant influence in multivariable analysis):

*Age* as a determinant is being discussed very controversially in the orthopaedic literature. Our results indicate a higher satisfaction of older patients, which is in accordance to the results of Chen et al. [8] and Kim et al. [9] for shoulder arthroplasties and rotator cuff repairs. Watson et al. [7] proposes that younger patients have higher demands and expectations of their shoulder and are therefore more easily dissatisfied with imperfect healing. There is, however also a number of publications, which deny a correlation between increasing age and higher patient satisfaction [12, 14, 19, 24, 25].

In our study, patients with a *private health care insurance* reached a significantly higher patient satisfaction than patients with a statutory health insurance. Furthermore, *patients receiving treatment by the chief of the department* seem to reach a higher satisfaction level than patients treated by (senior) consultants.

Our finding that *employed and retired patients* tend to be more satisfied than *unemployed and disabled patients* are in accordance with the results of the studies by Kim et al. [9] and Tashjian et al. [11]. Furthermore our data confirms the often reported correlation between *workers’ compensation claims* and a lower patient satisfaction [7–9, 13–17, 29, 30]. Also *postoperative anteversion/elevation* is an established determinant of the patient satisfaction in the orthopaedic literature [10, 12, 18, 19]. The results of our evaluation further support this correlation.

Also chronic alcohol abuse or a history of smoking (more than 10 pack-years) was associated with a low patient satisfaction. The later finding is contrary to the findings of Tashjian et al. [11]. A possible explanation for our result is the known impaired healing potential and the diminished collagen production of a chronic smoker [31–33].

Finally we were able to identify factors, which turned out to independently influence patients’ satisfaction (significant correlation in uni- and multivariable analysis):

As expected, remaining *pain* was identified to negatively influence patient satisfaction. This is in consensus to other published results for various shoulder interventions [8, 11, 12, 18, 19]. Our retrospective data analysis makes it however impossible to analyse the interesting question regarding the influence of peri- or immediate postoperative pain on the long-term outcome.

Furthermore the presence of *peri- or postoperative complications* affects patient satisfaction negatively, which is not unexpected, but to our knowledge so far unreported in the literature. In addition to that we analysed the influence of needed *revision surgery*, which negatively influenced patients satisfaction in the univariable analysis but showed only a trend (*p* = 0.062) in multivariable analysis.

*Postoperative abduction* was the only subvalue of the Constant Score, which turned out to independently positive influence patient satisfaction. This correlation seems to be unreported so far in orthopaedic literature and we should possibly focus even more on this determinant in postoperative rehabilitation.

The distinct correlation between patient satisfaction and the *postoperative subjective shoulder value* supports the validity of this analysis and clarifies the resemblance between these two subjective outcome parameters.

## Conclusion

This investigation establishes that the absence of perioperative complication, excellent control of postoperative pain, surprisingly active abduction in the scapular plane (not elevation!) are associated with high patient satisfaction after rotator cuff repair or shoulder arthroplasty. It is, however, particularly important, that factors such as retirement, light physical work, private health insurance status, non-smoking, absence of chronic alcohol abuse, older age or receiving treatment by the chief of the department, were correlated with higher patient satisfaction independent of the pathology and type of surgery. On the other hand our results document that in view of patient satisfaction the patients’ gender, marital status, dominance of the shoulder, nature of the injury, previous general operations, comorbidities, diabetes mellitus, psychotropic drugs or immunosuppressive medication are not associated with the ultimate subjective result. These findings may help to inform patients on their risks, to select patients for surgery and to focus peri- and postoperative treatment on the few modifiable factors identified to increase patient satisfaction.

## Abbreviations

BMI: Body Mass Index
COPD: Chronic obstructive pulmonary disease
CS: Constant Shoulder Score
HIV: Human immunodeficiency virus
n.s.: Not significant
Pat: Patients
SD: Standard deviation
SSV: Subjective Shoulder Value
